# Rheumatic Heart Disease and Contraception Use in Ethiopia: A Cross-Sectional Study

**DOI:** 10.7759/cureus.88541

**Published:** 2025-07-22

**Authors:** Samrawit A Zewde, Amha M Weldehana, Daniel A Zewde, Zelalem B Ayele, Shiferaw N Abebe

**Affiliations:** 1 Obstetrics and Gynecology, College of Health Science, Addis Ababa University, Addis Ababa, ETH; 2 Internal Medicine, College of Health Science, Addis Ababa University, Addis Ababa, ETH; 3 Internal Medicine, Saint Peter Referral Hospital, Addis Ababa, ETH

**Keywords:** contraception, preconception, pregnancy, rheumatic heart disease, women

## Abstract

Background

Women of reproductive age with rheumatic heart disease (RHD) face a high risk of cardiovascular complications during pregnancy. Consequently, preconception counseling and contraception are routinely recommended for all women of reproductive age. In Ethiopia, there are no data available regarding contraception coverage among women with RHD.

Objectives

The aims of this study were to examine the prevalence of contraceptive use, investigate the most common methods, and identify factors influencing contraception use among reproductive-age women with RHD in Ethiopia.

Methodology

The study included patients who were followed at a tertiary hospital between January 1, 2017, and October 30, 2018. A total of 359 women aged 15 to 49 years with RHD were randomly selected and evaluated using standardized questionnaires and chart reviews. Chi-square and logistic regression analyses were utilized to measure associations. A p-value of ≤0.05 was deemed significant, with a 95% confidence interval.

Results

The mean age was 30.96 ±7.84 years. Of the patients, 219 (61%) were unemployed, and only 92 (25%) had higher education. Of the 241 (67%) who had severe RHD, 205 (57%) had combined valve lesions, 76 (21.2%) had isolated mitral stenosis, and 62 (17.3%) had isolated mitral regurgitation. Of the total of 359 patients, 300 (83.6%) had symptoms; 241 (67.1%) were class IV per the modified World Health Organization (mWHO) cardiovascular risk assessment, and the rest, 118 (32.9%), were class III. A total of 139 (38.9%) and 112 (31.3%) were on warfarin and angiotensin-converting enzyme inhibitors, respectively. However, only 149 (41.5%) were on contraceptives, and among non-users, 226 (63%) reported no counseling. Injectables were the most used contraceptive, followed by intrauterine devices and oral pills. Marital status (OR: 3.26, 95% CI: 2.03-5.22, P < 0.001) and employment (OR: 3.82, 95% CI: 1.58-9.24, P = 0.003) were the only two statistically significant factors associated with contraception use.

Conclusion

Contraception use was low among women with RHD in Ethiopia. Patients with contraindications for pregnancy were not provided with adequate counseling and contraception. Additionally, those who were using contraception were on ineffective methods. Further action is needed to improve contraception use among women with RHD.

## Introduction

Rheumatic heart disease (RHD) is a common endemic cardiovascular disease in Sub-Saharan African (SSA) countries, with an estimated prevalence of 2.9% to 30% per 1,000 people [1,2]. According to the 2015 World Health Organization (WHO) reports, it was the ninth leading cause of death, affecting 40 million people worldwide and claiming 300,000 lives every year [3]. In Ethiopia, the prevalence is approximately 19 per 1,000 school children [4]. RHD commonly affects school children, adolescents, and young adults. Most of the affected groups are women of reproductive age, comprising two-thirds of the cases in a few studies conducted in Ethiopia [5]. As a result, women with RHD are at a high risk of cardiovascular complications and suffer from the poor outcomes associated with the disease. In Ethiopia, women give birth to five children per lifetime, and there are 267 maternal mortalities per 100,000 live births [6]. These figures are among the highest in the world, and cardiovascular diseases such as RHD contribute to the existing high mortality rates. Low socioeconomic status, poor sanitation, illiteracy, and unawareness are some of the determinants for its high prevalence. A mini-national survey conducted in Ethiopia in 2019 showed that 28.1% of women were using contraception nationally [7]. For the majority of RHD patients, irrespective of the type of valve lesion, contraception and prevention of pregnancy are recommended based on the severity of the disease [8]. However, no study investigated the prevalence of contraception use among RHD patients in Ethiopia. There are no data regarding the types of contraceptive methods commonly used, and factors affecting contraception use remain unknown. In this study, we investigated these unaddressed clinical questions to implement the right strategy to lower maternal and fetal deaths from RHD.

Many RHD patients in Sub-Saharan Africa do not have primary care physicians. Those with access to the health system are followed by a general doctor, internists, and, less commonly, a cardiologist. They rarely get referred to other specialties, such as obstetrics and gynecology, to address pregnancy and its complications during RHD management. As a result, providing healthcare that addresses all the unmet demands of women is crucial. One of the healthcare services needed is screening for pregnancy and providing preconception counseling and contraception during RHD management. Otherwise, pregnancy-related complications may occur and threaten the life of the mother and the fetus. Stenotic valve lesions, such as severe mitral stenosis (MS) and aortic stenosis (AS), can be symptomatic during pregnancy and cause acute decompensated heart failure. Unlike the two stenotic lesions, patients with mitral regurgitation (MR) and aortic regurgitation (AR) may tolerate pregnancy if their valve lesions are not severe enough or not associated with severe pulmonary hypertension [9]. Other conditions like severe pulmonary hypertension and reduced left ventricular function, absolute contraindications for pregnancy, may also be present and complicate stenotic or regurgitating valve abnormalities [10]. In these situations, pregnancy may need to be terminated to prevent anticipated severe complications such as acute decompensated heart failure, pulmonary edema, and refractory anasarca. In addition, medical therapies used in RHD may harm unborn fetuses. Warfarin, angiotensin receptor neprilysin inhibitors, angiotensin-converting enzyme inhibitors (ACEIs), or angiotensin receptor blockers (ARBs), which are common medications for heart failure management and thromboembolism prevention, are recommended to be avoided due to their teratogenic effects [11,12]. These risks underscore the importance of preconception counseling and contraception.

However, contraception use among the general population in Ethiopia is low, and RHD patients are no exception. In this study, we investigated the prevalence of contraception use and explored factors determining its use.

## Materials and methods

Study population and setting

The study was a cross-sectional, single-center, and hospital-based study conducted between January 1, 2017, and October 30, 2018. Based on prior research in Ethiopia, which indicated a 30% prevalence of contraception among patients with cardiovascular diseases, a sample size of 359 was calculated. Then, 359 women with RHD were randomly selected during the specified period from a cardiology clinic in Ethiopia's largest tertiary hospital. All women between 15 and 49 years of age and with a diagnosis of RHD were included. Women younger than 15 years and older than 49 years, those who had undergone hysterectomy, those who were pregnant at the time of the study, and those without RHD were excluded.

This study was approved by the Addis Ababa University, College of Health Science, Department of Obstetrics and Gynecology under a protocol number DRPC 2025/12/20. All procedures were conducted in accordance with the relevant ethical guidelines and regulations; Informed consent was obtained from all participants.

Data collection and variables

A standardized questionnaire was used to collect basic demographic information, the type of contraception used, current symptoms, and medications taken for RHD. The chart, including echocardiography results, was reviewed to confirm the presence of RHD and identify valvular abnormalities and complications. Based on the chart review, cardiovascular risk assessment was performed using a modified World Health Organization (mWHO) cardiovascular risk assessment tool, and patients were categorized into risk groups.

Data analysis

The data were analyzed using SPSS Version 25 (IBM Corp., Armonk, NY). Descriptive statistics, including frequency, mean, and standard deviation, were used to describe baseline characteristics. The chi-square or Fisher’s exact test and logistic regression with odds ratio were employed to measure associations between categorical variables. A p-value of ≤0.05 was considered significant for all measures of association with a 95% confidence interval.

Operational definitions

RHD was defined by echocardiography according to the World Heart Federation guidelines. All the patients included in the study underwent echocardiography performed by a cardiologist.

Contraception refers to any natural or artificial method to prevent pregnancy. Artificial methods include the use of injectables, implants, intrauterine devices (IUDs), oral contraceptive pills (OCPs), condoms, diaphragms, spermicides, and sterilization procedures, while natural methods include the calendar method, coitus interruptus, and breastfeeding.

The mWHO cardiovascular risk assessment tool is a risk-scoring model comprising four classes: class I, where there is no maternal risk; class II, mild risk; class III, significant risk requiring expert monitoring and follow-up; and class IV, extreme cardiovascular risk, where pregnancy is contraindicated.

## Results

Baseline characteristics

The mean age of the participants was 30.96±7.84 years. Among them, 225 (62.7%) were married, and 155 (43.2%) were nulliparous. Only 92 (25%) had higher levels of education, and 219 (61%) were unemployed. All other sociodemographic characteristics of the patients are given in Table 1.

**Table 1:** Baseline sociodemographic characteristics of women with rheumatic heart disease

Baseline variables	N (%), n= 359
Age (years)	
≤19	19 (5.3%)
20-24	63 (17.5%)
25-29	87 (24.2%)
30-34	68 (18.9%)
35-39	79 (22%)
≥40	43 (12%)
Marital status	
Single	134 (37.3%)
Married	225 (62.7%)
Religion	
Orthodox	221 (61.6%)
Protestant	79 (22%)
Muslim	59 (16.4%)
Educational level	
None	20 (5.6%)
Elementary	103 (28.7%)
High school	144 (40.1%)
Higher level	92 (25.6%)
Parity	
Nulliparous	155 (43.2%)
Primiparous	47 (13.1%)
Multiparous	146 (40.6%)
Grand multiparous	11 (3.1%)
Employment status	
Employed	140 (39%)
Unemployed	219 (61%)

Regarding RHD, 202 (57%) had the disease for over five years, and 205 (57%) had combined or multivalvular RHD. Isolated MS was the second most common valve abnormality, followed by MR. Isolated AS and AR were very rare. A total of 282 (78.6%) had an ejection fraction (EF) of ≥40%. Pulmonary hypertension was the most common complication observed in 332 (92%) patients, followed by 23 (6.4%) cases with recent acute decompensated heart failure. A total of 139 (38.9%) patients were using warfarin, and 112 (31.2%) were on ACEIs or ARBs. According to the modified WHO classification for maternal cardiovascular risk of complications, 241 (67.1%) of the patients were in the class IV risk group. All other clinical and echocardiographic details of the participants are given in Table 2.

**Table 2:** Baseline clinical and echocardiography characteristics of women with RHD ACEI, angiotensin-converting enzyme inhibitor; AR, aortic regurgitation; AS, aortic stenosis; MR, mitral regurgitation; MS, mitral stenosis; mWHO, modified World Health Organization; NYHA, New York Heart Association; RHD, rheumatic heart disease

Baseline variables	N (%), n= 359
Duration of RHD	
<5 years	156 (43.5%)
5-10 years	120 (33.4%)
>10 years	83 (23%)
Valve disease	
MS	76 (21.2%)
MR	62 (17.3%)
AS	6 (1.7%)
AR	10 (2.8%)
Combined	205 (57.1%)
Severity of valve disease	
Mild	27 (7.5%)
Moderate	91 (25.3%)
Severe	241 (67.1%)
Ejection fraction	
≤40%	77 (21.4%)
≥40%	282 (78.6%)
Complications of RHD	
Pulmonary hypertension	332 (92 %)
Heart failure	23 (6.4%)
Stroke	19 (5.3%)
Thromboembolism	1 (0.3%)
Others	1 (0.3%)
Cardiac surgery	
Yes	69 (19.2%)
No	290 (80.8%)
NYHA class	
I	59 (16.4%)
II	157 (43.7%)
III	89 (24.8%)
IV	54 (15%)
Medications	
Diuretics	92 (25.6%)
Digoxin	74(20.4%)
ACEI	112 (31.2%)
Warfarin	139 (38.9%)
mWHO class	
III	118 (32.9%)
IV	241 (67.1%)

Contraception use

**Figure 1:**
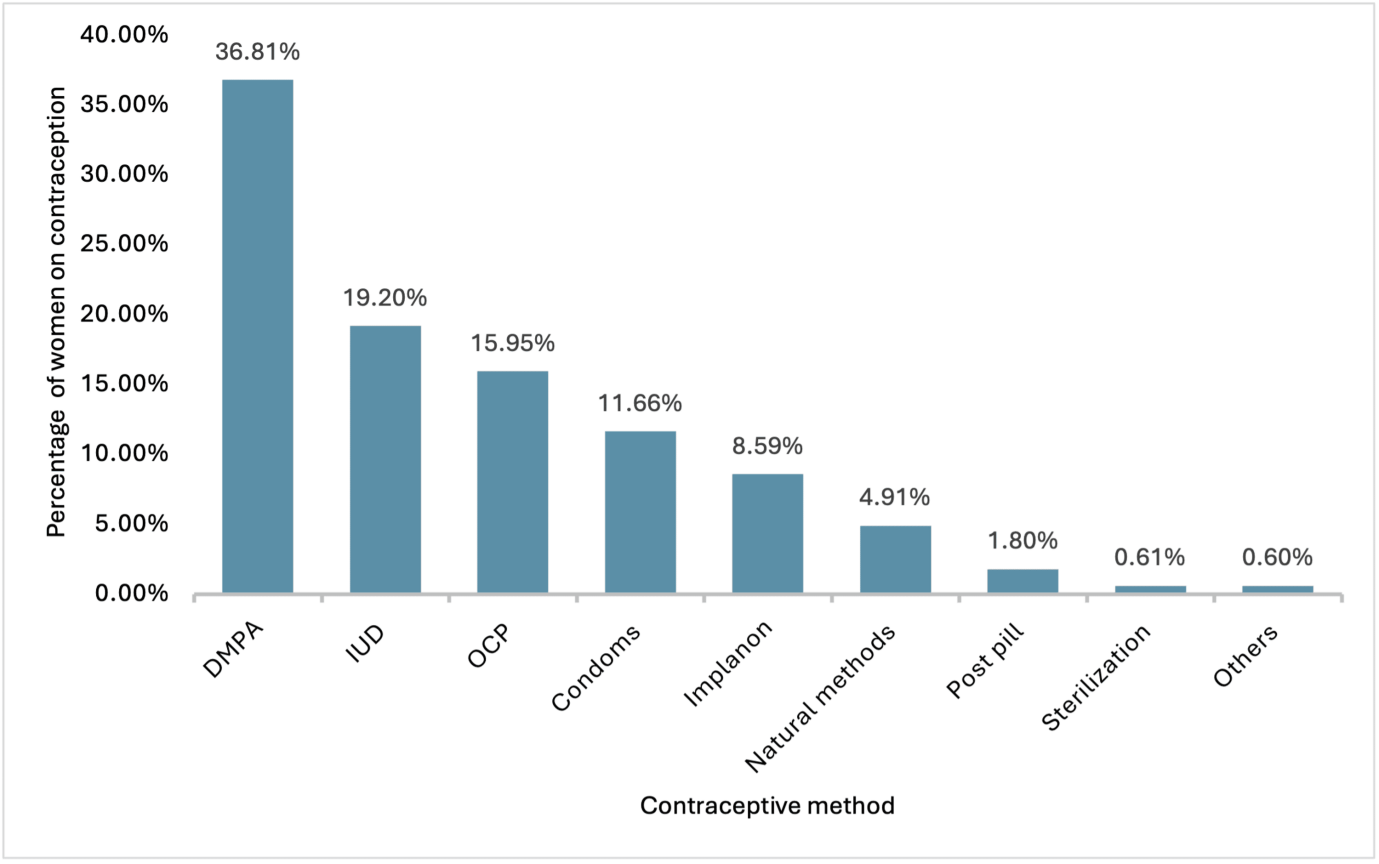
Distribution of contraceptive methods among women with rheumatic heart disease DMPA, depot medroxyprogesterone acetate; IUD, intrauterine device; OCP, oral contraceptive pills

Only 149 (41.5%) of the RHD patients were using either natural or artificial contraception methods. Among them, 132 (36.81%) used depot medroxyprogesterone acetate (DMPA) injection, followed by 69 (19.02%) using an IUD and 57 (15.95%) using oral OCPs (Figure 1).

Marital status (OR: 3.26, 95% CI: 2.03-5.22, P < 0.001) and employment (OR: 3.82 CI: 1.58-9.24, P = 0.003) were the two determinant factors observed to be associated with contraception use among women with RHD. Of the 144 people who were not using contraception, 27 (18.5%) reported that they had received counseling about contraception.

## Discussion

Preconception counseling and contraception for patients with RHD are often neglected in resource-limited settings despite their significant implications for maternal and fetal mortality. To our knowledge, no study has examined the clinical practice of preconception counseling and contraception use among patients with RHD in Ethiopia. Contraception data from other SSA countries is also extremely low. In general, Africa’s share of cardiovascular research output is only 3.1% globally, and our study aims to provide valuable cardiovascular data about the health systems operating in these impoverished regions [13]. Our study found that only 149 (41.5%) of women with RHD were using some form of contraception, while 241 (67.1%) had a class IV mWHO cardiovascular risk, where the pregnancy was contraindicated. Moreover, the study demonstrated the use of ineffective, sometimes contraindicated, contraceptive methods for high-risk patients.

Some baseline characteristics in the study highlighted the vulnerability of women to pregnancy-related complications in low-income countries. The mean age was 30 years, reflecting the young population commonly affected by RHD in Africa; 225 (62.7%) were already married, and 155 (43.2%) had no children. Additionally, only 92 (25%) had higher education, and 219 (61%) were unemployed. These features of poor socioeconomic status explain why 67% of the participants had severe RHD. Poor socioeconomic status and RHD are intertwined, with both being associated with poor maternal and fetal outcomes [14]. RHD is responsible for 34% of maternal deaths in Africa, and women in SSA countries have a 100-fold increased risk of death from pregnancy-related complications compared to women in developed countries [15]. The presence of longstanding RHD, severe and moderate stenotic valve lesions, a higher number of symptomatic patients, pulmonary hypertension, prior history of cardiac surgery, recent heart failure, and left ventricular dysfunction with EF ≤ 40% were some of the factors identified in our study that may contribute to the increased risk of cardiovascular complications during pregnancy. Additionally, one-third of the women were on warfarin and ACEIs/ARBs, which are teratogenic medications that should be avoided before and during pregnancy or used in conjunction with effective contraception methods.

Furthermore, our participants were evaluated using the standardized mWHO cardiovascular risk assessment tool; 241 (67%) were classified as class IV, where pregnancy was strictly contraindicated. The remaining participants were classified as class III, a high-risk group with poor outcomes that require close monitoring by an expert. We chose the mWHO risk assessment because of its familiarity with our health system and its advantages for congenital and acquired heart diseases, including RHD [16]. The mWHO or other cardiovascular risk assessment tools for cardiac patients during pregnancy have not been validated for the Ethiopian population.

Despite these alarming indicators of poor outcomes among patients with RHD, our study revealed that only 149 (41.5 %) were using some form of contraception. The remaining 210 (58.5%) were not using any form of contraception. Besides, only 27 (18.5%) out of the 210 patients not using contraception received counseling about it. A study conducted in Ethiopia and published in 2022 showed that 30% of women used contraception [17]. However, this study included women with all types of cardiovascular diseases and was not focused primarily on RHD. RHD is the most common cardiovascular disease among young women in Ethiopia. The prevalence of contraception use (41.5%) in our study is higher than some reported rates in SSA countries. For example, the REMEDY study of 12 African countries, Yemen, and India showed a 3.6% contraception use rate [18]. Similar studies from Tanzania and Uganda reported 7.1% and 14% contraception use, respectively [19,20]. The higher proportion of women using contraception in our study could be attributed to the fact that many of our study participants were from urban areas and had follow-up care at a tertiary-level hospital. These studies were all hospital-based, but the level of care provided by each hospital was not specified.

Although the proportion of women on contraception is higher compared to SSA studies, it is lower when compared to the rest of the world. A meta-analysis among women with cardiovascular diseases worldwide showed a 67% prevalence rate of contraception use [21]. A hospital-based study in New Zealand found that 38% of women with RHD were counseled and provided contraception [22]. These differences indicate that factors associated with contraception use are multifactorial and include patient and healthcare provider factors, as well as malfunctioning healthcare systems. In our study, we explored patient-related determinant factors and found that only marriage and employment had statistically significant associations with contraception use. Another study from Ethiopia showed that age, parity, education level, religion, socioeconomic status, and number of living children were important factors for the use of modern contraception among women in Ethiopia [23]. Contrary to this study, factors such as education level, religion, and parity were not associated with contraception use in our study.

Lastly, our study identified DMPA as the most common form of contraception used among RHD women, followed by IUD and OCP. Few patients used post-pills and sterilization methods. The most effective contraceptive methods, such as IUD and Implanon, were the second and fifth choices, respectively [24]. Both have 99% effectiveness in preventing pregnancy. Injectables such as DMPA are 94% effective and require injections every three months. The popularity of its use might be due to the less frequent hospital visits needed and the reversibility of fertility upon discontinuation. On the other hand, the use of OCPs prevents 91% of pregnancies and increases the risk of thromboembolism, especially among patients with mechanical valves [25]. Considering the use of these less ineffective contraceptive methods, the proportion of patients using effective contraceptive methods is likely lower than reported. In general, there are no head-to-head comparisons for effective contraception methods for women with cardiovascular diseases. However, long-term contraception methods with less risk of thromboembolism, such as IUDs, are recommended [24].

Limitations of the study

The study was a single-center, hospital-based cross-sectional study. Hospital-based studies may have limitations in estimating the actual prevalence. Additionally, the patients who were followed at the tertiary hospital were a selected group of patients from urban areas with better access to contraception and awareness of pregnancy complications. These factors might have contributed to the relatively higher number of contraceptive users compared to other studies conducted in Africa. On the other hand, the study is limited in characterizing the knowledge, attitude, and beliefs of patients included in the study.

## Conclusions

Contraception use was low among women with RHD in Ethiopia. Appropriate preconception counseling and contraception services were not provided for women with high-risk conditions or contraindications for pregnancy and those taking medications such as warfarin and ACEIs/ARBs. The use of ineffective contraceptive options was also prevalent. These findings indicate that the current Ethiopian health system is not functioning well to address one of its major development plans, such as reducing maternal and fetal mortality. Appropriate referral and multidisciplinary teamwork involving allied health professionals may mitigate the current low and inappropriate contraception use among women with RHD.
